# Polymorphisms of the *TUB* Gene Are Associated with Body Composition and Eating Behavior in Middle-Aged Women

**DOI:** 10.1371/journal.pone.0001405

**Published:** 2008-01-09

**Authors:** Jana V. van Vliet-Ostaptchouk, N. Charlotte Onland-Moret, Ronit Shiri-Sverdlov, Patrick J. J. van Gorp, Anne Custers, Petra H. M. Peeters, Cisca Wijmenga, Marten H. Hofker, Yvonne T. van der Schouw

**Affiliations:** 1 Department of Pathology and Laboratory Medicine, University Medical Center Groningen, University of Groningen, Groningen, The Netherlands; 2 Department of Molecular Genetics, Maastricht University, Maastricht, The Netherlands; 3 DBG-Department of Medical Genetics, University Medical Center Utrecht, Utrecht, The Netherlands; 4 Julius Center for Health Sciences and Primary Care, University Medical Center Utrecht, Utrecht, The Netherlands; 5 Department of Genetics, University Medical Center Groningen, University of Groningen, Groningen, The Netherlands; University College London, United Kingdom

## Abstract

**Background:**

The *TUB* gene, encoding an evolutionary conserved protein, is highly expressed in the hypothalamus and might act as a transcription factor. Mutations in *TUB* cause late-onset obesity, insulin-resistance and neurosensory deficits in mice. An association of common variants in the *TUB* gene with body weight in humans has been reported.

**Methods/Findings:**

The aim was to investigate the relationship of single nucleotide polymorphisms (SNPs) of the *TUB* gene (rs2272382, rs2272383 and rs1528133) with both anthropometry and self-reported macronutrient intake from a validated food frequency questionnaire. These associations were studied in a population-based, cross-sectional study of 1680 middle-aged Dutch women, using linear regression analysis. The minor allele *C* of the rs1528133 SNP was significantly associated with increased weight (+1.88 kg, *P* = 0.022) and BMI (+0.56 units, *P* = 0.05). Compared with non-carriers, both *AG* heterozygotes and *AA* homozygotes of the rs2272382 SNP derived less energy from fat (*AG*: −0.55±0.28%, *P* = 0.05, *AA*: −0.95±0.48%, *P* = 0.047). However, both genotypes were associated with an increased energy intake from carbohydrates (0.69±0.33%, *P* = 0.04 and 1.68±0.56%, *P* = 0.003, respectively), mainly because of a higher consumption of mono- and disaccharides. Both these SNPs, rs2272382 and rs1528133, were also associated with a higher glycemic load in the diet. The glycemic load was higher among those with *AG* and *AA* genotypes for the variant rs2272382 than among the wild types (+1.49 (95% CI: −0.27–3.24) and +3.89 (95% CI: 0.94–6.85) units, respectively). Carriers of the minor allele *C* of rs1528133 were associated with an increased glycemic load of 1.85 units compared with non-carriers.

**Conclusions:**

Genetic variation of the *TUB* gene was associated with both body composition and macronutrient intake, suggesting that *TUB* might influence eating behavior.

## Introduction

The hypothalamus plays a central role in the control of energy balance and the regulation of body weight and food intake [1]–[3]. The tubby protein is highly expressed in the paraventricular (PVN), ventromedial (VMH), and arcuate nuclei (ARC) of the hypothalamus that regulate satiety and appetite [4]–[6]. Loss-of-function mutations in tubby result in late-onset obesity, insulin resistance and neurosensory deficits in mice [7]. Further investigations showed that the expression of different neuropeptides was altered. Lower levels of agouti-related protein (AGRP) and proopiomelanocortin (POMC) mRNA levels in ARC were paralleled by increased levels of neuropeptide Y (NPY) in the dorsomedial-ventromedial hypothalamus (DMH/VMH) as well as preproorexin mRNA in the lateral hypothalamus (LHA) in mature and juvenile tubby mice [8]–[10]. Such multiple derangements in the neural system lead to hyperphagia in the adult animals [8].

Tubby homologs are highly conserved among vertebrate genomes [11] and in *C. elegans* the important role of the tubby ortholog, tub-1, in fat storage regulation has been shown [12]–[14]. Several studies have suggested that tubby may function as a transcription factor and/or as an adaptor molecule for downstream signaling of insulin and/or G-protein-coupled receptors [15]–[17].

Previously, we reported a significant association between variants in the *TUB* gene and body mass index (BMI) in a Dutch population of type 2 diabetes patients [18]. The minor alleles of single nucleotide polymorphisms (SNPs) rs2272382, rs2272383 and rs1528133 were associated with an average of 1.5 kg/m^2^ higher BMI, and were 1.3 times more frequent among obese people (BMI> = 30 kg/m^2^) than lean individuals (BMI<25 kg/m^2^).

In order to ascertain the validity of our previous results in a population-based sample, and to explore whether macronutrient intake may be involved in the development of overweight and/or obesity, the present study investigated the effects of genetic variants in the *TUB* gene on food intake and body composition in middle-age Dutch women.

## Materials and Methods

### Subjects

The women included in this study are Dutch participants in the European Prospective Investigation into Cancer and Nutrition (EPIC), conducted in Utrecht, the Netherlands (Prospect-EPIC) [19]. Between 1993 and 1997, 17,357 women aged 49–70 years and residing in or near Utrecht were recruited through a regional, population-based, breast cancer screening program. All the women signed written informed consent and the study was approved by the Institutional Review Board of the University Medical Center Utrecht.

At recruitment, each participant filled out a general questionnaire on lifestyle factors, gynecological and obstetric history, and past and current morbidity, as well as a validated semi-quantitative food frequency questionnaire (FFQ) with the aim of capturing the habitual diet during the year preceding enrolment. In addition, pulse rate, blood pressure and anthropometric measurements were taken and a blood sample was donated and stored at −196°C. A random sample of 1736 (10%) women was taken for biochemical analyses. Buffy coat samples were missing for 56 women, so the study population comprised 1680 women.

### Anthropometry variables

Body height was measured to the nearest 0.5 cm with a wall mounted stadiometer (Lameris, Utrecht, the Netherlands). Body weight was measured in light indoor clothing without shoes to the nearest 0.5 kg with a floor scale (Seca, Atlanta, GA, USA). Body mass index (BMI) was calculated as weight divided by height squared (kg/m^2^). Waist and hip circumferences were measured to the nearest 0.5 cm with a standard household tape measure, and the waist-to-hip ratio was calculated.

### Food frequency questionnaire

The FFQ contains questions on the usual frequency of consumption of 77 main food items during the year preceding enrollment. Further information was sought on consumption frequency for different sub-items, preparation methods, and additions. Color photographs were used to estimate portion size for 28 food items. Overall, the questionnaire allows the estimation of the average daily consumption of 178 foods, by asking about sub-items for several foods, like fruit and vegetables, in additional questions. The FFQ was validated in pilot studies prior to the start of our study [20]. We calculated glycemic load by multiplying the glycemic index of a food with its carbohydrate content, then multiplied this value with the frequency of consumption of this food, and summed the values over all food items [21]. Glycemic load thus represents both the quality and quantity of carbohydrates and the interaction between the two. Each unit of dietary glycemic load represents the equivalent of 1 g carbohydrate from glucose. The overall glycemic index of a woman's diet was calculated by dividing the dietary glycemic load by the total amount of carbohydrate consumed [21]. Such expression of dietary glycemic index per gram of carbohydrate thus reflects the overall quality of the daily carbohydrate intake.

### Genotyping

DNA was extracted from whole blood using the QIAamp® Blood Kit (Qiagen Inc., Valencia, CA, USA). SNPs rs2272382, rs2272383 and rs1528133, previously reported to be associated with BMI (18), were genotyped using Taqman assay-on-demand (Applied Biosystems, Foster City, CA, USA). Assays were performed according to the manufacturer's specifications (assays C_86243_10, C_271936_1, C_9597097_1, respectively). The genotypes were analyzed using a TaqMan 7900HT (Applied Biosystems, Foster City, CA, USA). The DNA samples were processed in 384-well plates, each containing 4 negative controls and 12 genotyping controls (four duplicates of three different samples obtained from the Centre d'Etude du Polymorphisme Humain or CEPH). The genotype success rates were always >95% (95.7% for rs2272382, 96.5% for rs2272383 and 96.4% for rs1528133). The SNPs did not significantly deviate from Hardy-Weinberg equilibrium (χ^2^ = 0.41, *p* = 0.52 for rs2272382, χ^2^ = 1.38, *p* = 0.24 for rs2272383 and χ^2^ = 0.24, *p* = 0.63 for rs1528133). There were no discordances in the genotypes of any of the CEPH samples.

### Statistical analysis

All statistical analyses were performed using the SPSS program, version 13.0 for Windows (SPSS, Chicago, IL, USA). Means with standard deviation, median and range (for the not-normally distributed characteristics) or frequencies (where appropriate) of baseline characteristics were presented for the entire population. The genotype frequencies were tested for Hardy-Weinberg equilibrium using a χ^2^ test with 1 df. The association between genotypes of SNPs and the cohort's anthropometrical characteristics and macronutrient intakes were calculated using a linear regression model. Individuals homozygous for the common allele served as the reference group. Linkage disequilibrium (LD) among SNPs was computed using Haploview program, version 4.0RC1 [22].

## Results

**Figure 1:**
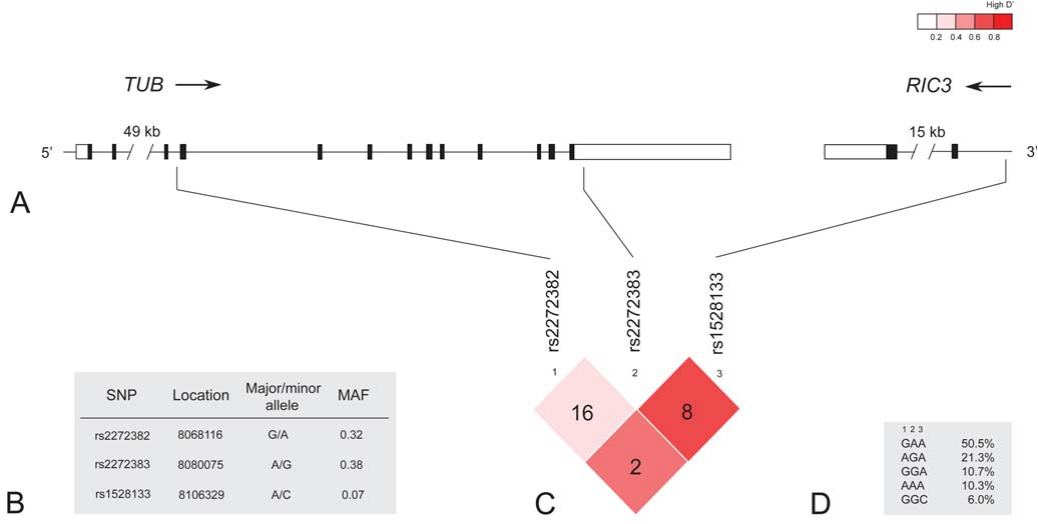
Gene structure, location and characteristics of the genotyped SNPs, linkage disequilibrium (LD) among SNPs and the haplotype structure. A: The upper panel shows the 90-kb region of chromosome 11p15.5 containing the *TUB* gene and the 3′ flanking *RIC3* gene. Coding sequences (black), UTRs (white) and introns are indicated. B: Location (according to the NCBI 35 release), the alleles and the minor allele frequencies (MAF) of the SNPs. C: The pairwise LD plot, calculated as r^2^, among SNPs, with red colour indicating increasing level of LD as measured by D'. D: The three genotyped SNPs resulted in five common haplotypes.

We genotyped the three SNPs (rs2272382, rs2272383 and rs1528133) reported to be significantly associated with BMI in our previous study [18]. **Figure 1** shows the structure of the *TUB* gene and its 3′ flanking region and the position of the genotyped SNPs. The allele frequencies of the variants as well as the pairwise linkage disequilibrium among SNPs were comparable with those reported by Shiri-Sverdlov *et al* (18) (Figure 1B,C). The haplotype analysis of the three genotyped SNPs revealed five common haplotypes (Figure 1,D).

Food intake and anthropometrical characteristics of the 1680 women are shown in **Table 1**. First, we investigated the association of genetic variants of the *TUB* gene with different measures of body composition. The variant rs1528133 was significantly associated with weight and BMI (**Table 2**). The carriers of the minor allele C had an average increase in weight of 1.88 kg (95% CI: 0.27–3.48) and of 0.56 units in BMI (95% CI: 0.0–1.12) compared with non-carriers. The same trend was observed for waist and hip circumferences, but it was less pronounced.

**Table 1:**
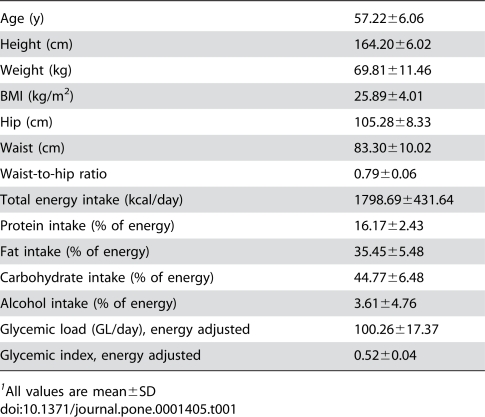
Anthropometrical and food intake characteristics of 1680 women from the EPIC Cohort^1^.

1 All values are mean±SD

**Table 2:** Anthropometrical characteristics of 1680 women from the EPIC study by genotype for rs1528133 SNP in the *TUB* gene.

	rs1528133			
	AA (*n* = 1392)	AC+CC (*n* = 217+10)		
	Mean±SD	β	95% CI	*p*-value
Weight (kg)	69.56±0.30	1.88	0.27–3.48	0.022
Waist (cm)	83.13±0.26	1.23	−0.17–2.64	0.09
Hip (cm)	105.13±0.22	1.10	−0.07–2.27	0.06
BMI (kg/m^2^)	25.81±0.11	0.56	0.0–1.12	0.050
Waist-to-hip ratio	0.79±0.0	0.0	0.0–0.01	0.40

**Table 3** shows intake of macronutrients, expressed as a percentage of total energy intake, according to the *TUB* gene SNPs. The rs2272382 variant was significantly associated with fat and carbohydrate intake. In comparison with non-carriers, the percentage of energy derived from fat was 0.55% (95% CI: −1.10–0.00) less in heterozygotes and 0.95% (95% CI: −1.88–−0.01) less in homozygotes for the minor allele *A*. The observed differences were mainly due to a lower percentage of energy derived from saturated and monounsaturated fats. However, both *AG-* and *AA-*genotypes showed significant association with higher carbohydrate intake compared with *GG*-genotype (0.69% (95% CI: 0.03–1.34) and 1.68% (95% CI: 0.58–2.78), respectively), mainly because of a higher consumption of mono- and disaccharides. A similar but weaker pattern of association with a lower percentage of energy from fat and higher percentage of energy from carbohydrates was found for the minor allele of rs2272383. No differences were observed in total energy intake and the intake of other macronutrients (data not shown). Adjustment for age and BMI did not alter the results (data not shown).

**Table 3:** Association of dietary phenotypes with the single nucleotide polymorphisms in the *TUB* gene.

	rs2272382						
	GG ( *n* = 736)	AG (*n* = 712)			AA (*n* = 160)		
Macronutrient intake (% of energy)	Mean intake±SD	β	95% CI	*p*-value	β	95% CI	*p*-value
Protein intake	16.28±0.09	−0.27	−0.52–−0.03	**0.028**	0.04	−0.37–0.46	**0.84**
Fat intake	35.77±0.19	−0.55	−1.10–0.00	**0.05**	−0.95	−1.88–−0.01	**0.047**
Saturated fat	15.44±0.10	−0.32	−0.61–−0.04	**0.027**	−0.38	−0.86–0.10	**0.12**
Monounsaturated fat	13.30±0.09	−0.20	−0.45–0.05	**0.11**	−0.54	−0.95–−0.12	**0.011**
Polyunsaturated fat	6.72±0.06	−0.03	−0.21–0.16	**0.78**	−0.02	−0.33–0.28	**0.88**
Carbohydrates intake	44.32±0.23	0.69	0.03–1.34	**0.040**	1.68	0.58–2.78	**0.003**
Mono- and disaccharides	23.68±0.21	0.86	0.26–1.47	**0.005**	1.08	0.06–2.10	**0.038**
Polysaccharides	20.59±0.14	−0.17	−0.58–0.24	**0.42**	0.60	−0.09–1.29	**0.09**
Alcohol intake	3.62±0.17	0.14	−0.34–0.62	**0.58**	−0.77	−1.58–0.04	**0.06**

	rs2272383						
	AA (*n* = 602)	AG (*n* = 790)			GG (*n* = 229)		
Macronutrient intake (% of energy)	Mean intake±SD	β	95% CI	*p*-value	β	95% CI	*p*-value
Protein intake	16.17±0.09	−0.01	−0.26–0.24	**0.94**	0.00	−0.37–0.37	**1.00**
Fat intake	35.72±0.21	−0.40	−0.97–0.16	**0.16**	−0.60	−1.43–0.23	**0.15**
Saturated fat	15.45±0.11	−0.32	−0.61–−0.02	**0.035**	−0.26	−0.69–0.17	**0.24**
Monounsaturated fat	13.31±0.09	−0.20	−0.46–0.05	**0.11**	−0.36	−0.73–0.01	**0.05**
Polyunsaturated fat	6.65±0.07	0.12	−0.07–0.31	**0.20**	0.01	−0.26–0.29	**0.92**
Carbohydrates intake	44.53±0.25	0.31	−0.36–0.98	**0.36**	0.71	−0.27–1.69	**0.16**
Mono- and disaccharides	23.95±0.23	0.26	−0.36–0.88	**0.42**	0.58	−0.32–1.49	**0.21**
Polysaccharides	20.53±0.16	0.06	−0.37–0.48	**0.79**	0.13	−0.49–0.74	**0.69**
Alcohol intake	3.57±0.19	0.10	−0.39–0.59	**0.69**	−0.11	−0.83–0.61	**0.77**

	rs1528133			
	AA (*n* = 1392)	AA+CC (*n* = 217+10)		
Macronutrient intake (% of energy)	Mean intake±SD	β	95% CI	*p*-value
Protein intake	16.14±0.06	0.20	−0.14±0.55	**0.24**
Fat intake	35.48±0.14	−0.25	−1.02±0.52	**0.52**
Saturated fat	15.26±0.07	0.05	−0.35±0.45	**0.81**
Monounsaturated fat	13.19±0.06	−0.21	−0.56±0.13	**0.23**
Polyunsaturated fat	6.72±0.05	−0.09	−0.34±0.17	**0.50**
Carbohydrates intake	44.75±0.17	0.17	−0.74±1.08	**0.72**
Mono- and disaccharides	24.12±0.16	0.15	−0.69±1.00	**0.72**
Polysaccharides	20.57±0.11	0.01	−0.57±0.58	**0.97**
Alcohol intake	3.62±0.13	−0.12	−0.79±0.55	**0.72**

The analysis of association between SNPs of the *TUB* gene and glycemic load (GL) and glycemic index (GI) is shown in **Table 4**. Two SNPs, rs2272382 and rs1528133, were associated with a higher GL in the diet. The GL was higher among those with *AG* and *AA* genotypes for the variant rs2272382 than among the wild types (+1.49 (95% CI: −0.27–3.24) and +3.89 (95% CI: 0.94–6.85) units, respectively). Carriers of the minor allele *C* of rs1528133 were also associated with an increased GL of 1.85 units compared with non-carriers. We observed no association of the *TUB* gene SNPs with glycemic index.

**Table 4:** Association of glycemic load (GL) and glycemic index (GI) with the single nucleotide polymorphisms in the *TUB* gene.

Glycemic load							
SNP	Wild type	Heterozygote			Minor homozygote		
	Mean intake±SD	β	95% CI	*p*-value	β	95% CI	*p*-value
rs2272382	99.26±0.61	1.49	−0.27–3.24	**0.097**	3.89	0.94–6.85	**0.010**
rs2272383	99.62±0.68	0.97	−0.83–2.77	**0.29**	1.37	−1.26–4.00	**0.31**
rs1528133 (hetero- and minor homo-zygotes combined)	99.33±0.64	1.85	0.13–3.57	**0.035**	-	-	-

Glycemic index							
SNP	Wild type	Heterozygote			Minor homozygote		
	Mean intake±SD	β	95% CI	*p*-value	β	95% CI	*p*-value
rs2272382	0.52±0.0	0.0	0.0–0.0	**0.89**	0.0	0.0–0.0	**0.91**
rs2272383	0.52±0.0	0.0	0.0–0.0	**0.64**	0.0	0.0–0.0	**0.62**
rs1528133 (hetero- and minor homo-zygotes combined)	0.52±0.0	0.0	0.0–0.0	**0.75**	-	-	**-**

## Discussion

This study shows association between polymorphisms in the *TUB* gene and body composition, fat and carbohydrate intake, and glycemic load in a Dutch female population.

In a previous study, we found that common variants of the *TUB* gene can influence body weight and contribute to obesity in diabetics [18]. In this study, we have confirmed that the minor allele for rs1528133 is significantly associated with increased weight and BMI, and thus show that the relation of *TUB* with body composition can be extended to a general population.

In addition, we found a significant association between rs1528133 and specifically the intake of food with a higher glycemic load. Variant rs2272382 was also related to the differences in macronutrients intake and glycemic load: individuals carrying the minor allele *A* of rs2272382 derived significantly less of their energy from fat, especially saturated and monounsaturated fat, and more from carbohydrates, in particular mono- and disaccharides, than carriers of the major *G* allele. Moreover, subjects carrying both the *AA* and *AG* genotype showed an association with higher glycemic load. These observed differences in macronutrient intakes were not related with the total energy intake.

Both SNPs rs1528133 and rs2272382 are located in noncoding regions and thus are not likely to be functional variants. The rs1528133 polymorphism is located in the intron of the *RIC3* gene, which is flanking the 3′ end of *TUB*. *RIC3* is playing a role in neurotransmission [23] and can not, therefore, be excluded as a candidate gene for obesity. However, the observed association is more likely to be due to the strong linkage disequilibrium of rs1528133 with SNPs in the highly conserved 3′ end of the *TUB* gene (Figure S1), since the mutations in this part of *TUB* are known to cause obesity in tubby mice [6], [24]. In addition, we explored the pairwise LD between the rs2272382, rs2272383 and rs1528133 variants of *TUB* and other SNPs in the gene using HapMap CEU data. The analysis did not reveal any linkage of these SNPs with the known coding polymorphisms. Hence, it remains unclear whether the associated SNPs are in the LD with a yet unknown causal variant or are themselves functional by affecting the gene expression. To date, the difference in the tubby protein expression between obese and normal weight individuals was lately reported [25]. Altogether, all of this implies the importance of further investigation into the *TUB* role in human obesity.

The strengths of the current study include its large sample size and its population-based design, combined with the extensive dietary information collected through a validated instrument. In spite of observations that overweight individuals tend to underestimate their food intake [26]–[28], we did find associations with fat and carbohydrate intake, and glycemic load. Nevertheless, we cannot exclude the possibility that small effects on total energy intake may have been diluted to null because of this underestimation.

It needs to be noted that the current study was restricted to women. However, in the previous study we observed a statistically significant association between the *TUB* gene variants and BMI in both women and men (Shiri-Sverdlov, unpublished data).

The findings of the present study reveal that the carriers of the *TUB* variants who have an increased BMI and body weight consumed a diet high in carbohydrates, in particular because of their intake of mono- and disaccharides. Also, the positive association between these variants and glycemic load compared with non-carriers suggests that the effect of the *TUB* gene on body composition might be due to the increased intake of simple carbohydrates.

Recently, some evidence has accumulated that daily caloric intake and the selection of macronutrients in the diet are partly inherited [29]–[31]. It has been suggested that neuropeptides known to regulate eating behavior might influence the intake of specific nutrients [32] and effects of polymorphisms in these genes on macronutrient intake in humans has been reported [33]–[36]. Recent studies have shown that a polymorphism of the *serotonin 5-HT receptor (5HT2A)* gene is a risk factor for anorexia nervosa [37] and that it also has an effect on food intake. The risk allele *A* was associated with decreased fat intake in young people [34] and the *G* allele was related to increased carbohydrate and alcohol intake in obese individuals [35]. Loos *et al* have described the effect of genetic variants in the *AGRP* gene, associated with leanness, on fat and carbohydrate intakes [36].

The *TUB* is highly expressed in the paraventricular (PVN), ventromedial (VMH), and arcuate nuclei (ARC) of the hypothalamus that regulate satiety and appetite [4]–[6], [38], [39]. The exact mechanism linking the loss of the *TUB* function in the tubby mice, and their adult-onset obesity is still not clear. However, alteration of the expression of different neuropeptides in mutant animals has been observed, such as upregulation of neuropeptide Y (NPY) by ∼30-fold in the dorsomedial-ventromedial hypothalamus (DMH/VMH) of mature tubby mice [9], as well as altered expression of the agouti-related protein (AGRP) in the ARC nuclei of the hypothalamus [8]. Recently, it was shown that preproorexin mRNA in the lateral hypothalamus (LHA) of the juvenile non-obese tubby mice was upregulated by ∼60-fold, which resulted in increased orexin A protein levels [10]. Such disturbance in the expression of different neuropeptides, caused by loss-of-function mutations in *TUB*, suggests it plays an important role in the system controlling the appetite and feeding behavior. Hence, we hypothesized that *TUB* plays a role in modulating food intake.

**Figure 2:**
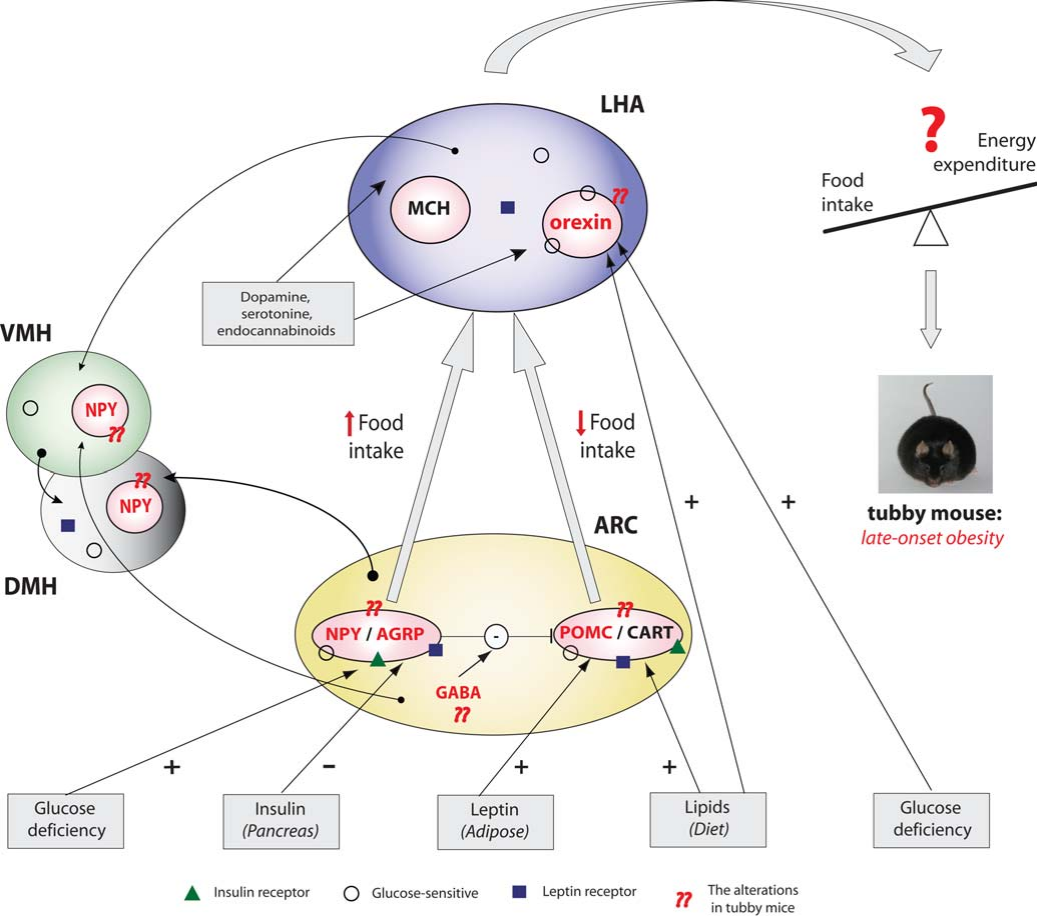
The interactions between the neuropeptides in the hypothalamus regulating energy homeostasis and its alteration in tubby mice. Signals related to diet and circulating nutrients (shown at the bottom), which stimulate (+) or inhibit (−) the production of peptides, are received by the various hormonal receptors in the ARC nuclei, which contain NPY/AGRP- and POMC/CART-producing groups of cells. The activation of NPY/AGRP neurons promotes food intake, whereas POMC/CART neurons have the opposite effect. Both signals project onto LHA neurons that express MCH and orexin (important stimulators of food intake), which are also mediated by dopamine, serotonin and endocannabinoids. The loss of the *TUB* function in tubby mice causes alteration in the expression of different neuropeptides, such as AGRP, NPY and POMC in ARC, as well as upregulation of NPY by ∼30-fold in DMH/VMH and orexin by ∼60-fold in LHA (highlighted in red). The image of tubby mice was provided by J.Naggert and P. Nishina (Nat. Genet. 39,149; 2007). Abbreviations: AGRP, agouti-related protein; ARC, arcuate nucleus; CART, cocaine–amphetamine-regulated transcript; DMH and VMH, dorsomedial and ventromedial hypothalamus; LHA, lateral hypothalamus; MCH, melanin-concentrating hormone; NPY neuropeptide Y; POMC, pro-opiomelanocortin.

The results of the present study show, for the first time, that genetic variation of the *TUB* gene might influence food preferences in humans. To our knowledge, no explicit studies of the “tub effect” on feeding behavior in animals have been performed either, except for the reported hyperphagia in the adult tubby mice compared to their normal littermates [8]. Thus, it is unknown how *TUB* can stimulate the selective intake of macronutrients. Nevertheless, there are several possible mechanisms that might explain our observations (**Figure 2**). First, *TUB* may exert its effect through NPY, which is known to be positively linked to an animal's voluntary selection of carbohydrates and it has been suggested it plays a role in the signaling of deficiency of glucose availability, -stores or -utilization [40]. Second, *TUB* may influence the intake of fat through AGRP, shown to increase the preferences towards a high-fat diet in animals [41], [42]. Third, the possible interconnections between *TUB* and orexin, which is highly responsive to changes in circulating and dietary nutrients [40], are indicated by its high upregulation in the young tubby mice. Moreover, orexin was reported to interact with serotonin (5-HT) in appetite regulation [43]. Interestingly, tub has also been shown to translocate from the plasma membrane to the nucleus after serotonin activation of 5-HT2c receptors [15], which could point to a function of tub as a downstream effector of these receptors.

An important question remains how a high-carbohydrate diet can influence body weight. Observational studies have suggested that the type of carbohydrates consumed may be related to body weight [44]–[46], and this was supported by the results of a recent trial in which both high-protein and low- glycemic index (GI) regimens led to a greater loss of body fat, but cardiovascular risk reduction was optimized by a high-carbohydrate, low-GI diet [47].

The importance of glycemic load (GL) compared with glycemic index (GI) was emphasized before [48]. GI indicates carbohydrate quality, and ranks how rapidly a particular food turns into sugar, whereas GL reflects both the quality and quantity of carbohydrate in the diet. Therefore, GL may better characterize a food's physiological effect than the amount of carbohydrates or its GI [49]. Based on the results of our study, it can be speculated that *TUB* may represent a genetic component influencing eating preferences towards the high-GL diet, which results in the increased weight and BMI. Additional investigations should be carried out in other populations to understand the exact mechanism of *TUB* on food intake.

In conclusion, this study has shown for the first time that polymorphisms of the *TUB* gene, previously shown to influence body weight, are also associated with differences in macronutrients intake and glycemic load and could thus be related to eating behavior in humans. However, replication studies in other populations are necessary to confirm our findings. Further functional studies of *TUB* could also clarify the observed effects on food intake and reveal the physiological role of that gene in the central regulation of body weight and food intake.

## Supporting Information

Figure S1 Detailed picture of TUB gene and it's 3′ flanking region and the location of all SNPs currently to be found in HapMap in this region (http://www.hapmap.org). The pairwise linkage disequilibrium (LD) plot, calculated as r2, among SNPs is shown, with red colour indicating increasing level of LD as measured by D'. The red boxes indicate SNPs genotyped in this study.
(11.07 MB EPS)
